# Familial Alzheimer’s Disease Lymphocytes Respond Differently Than Sporadic Cells to Oxidative Stress: Upregulated p53-p21 Signaling Linked with Presenilin 1 Mutants

**DOI:** 10.1007/s12035-016-0105-y

**Published:** 2016-09-19

**Authors:** Joanna Wojsiat, Katarzyna Laskowska-Kaszub, Carolina Alquézar, Emilia Białopiotrowicz, Noemi Esteras, Mykola Zdioruk, Angeles Martin-Requero, Urszula Wojda

**Affiliations:** 1 Laboratory of Preclinical Testing of Higher Standard, Nencki Institute of Experimental Biology, Pasteur 3, 02-093 Warsaw, Poland; 2 Department of Cellular and Molecular Medicine, Centro de Investigaciones Biológicas (CSIC), Ramiro de Maeztu 9, 28040 Madrid, Spain; 3 CIBER de Enfermedades Raras (CIBERER), 28040 Madrid, Spain

**Keywords:** Familial Alzheimer’s disease, Sporadic Alzheimer’s disease, Presenilin, Oxidative stress, Apoptosis, 2-deoxy-D-ribose, Lymphocytes, p53, p21, Mitochondrial membrane potential

## Abstract

Familial (FAD) and sporadic (SAD) Alzheimer’s disease do not share all pathomechanisms, but knowledge on their molecular differences is limited. We previously reported that cell cycle control distinguishes lymphocytes from SAD and FAD patients. Significant differences were found in p21 levels of SAD compared to FAD lymphocytes. Since p21 can also regulate apoptosis, the aim of this study was to compare the response of FAD and SAD lymphocytes to oxidative stress like 2-deoxy-D-ribose (2dRib) treatment and to investigate the role of p21 levels in this response. We report that FAD cells bearing seven different PS1 mutations are more resistant to 2dRib-induced cell death than control or SAD cells: FAD cells showed a lower apoptosis rate and a lower depolarization of the mitochondrial membrane. Despite that basal p21 cellular content was lower in FAD than in SAD cells, in response to 2dRib, p21 mRNA and protein levels significantly increased in FAD cells. Moreover, we found a higher cytosolic accumulation of p21 in FAD cells. The transcriptional activation of p21 was shown to be dependent on p53, as it can be blocked by PFT-α, and correlated with the increased phosphorylation of p53 at Serine 15. Our results suggest that in FAD lymphocytes, the p53-mediated increase in p21 transcription, together with a shift in the nucleocytoplasmic localization of p21, confers a survival advantage against 2dRib-induced apoptosis. This compensatory mechanism is absent in SAD cells. Thus, therapeutic and diagnostic designs should take into account possible differential apoptotic responses in SAD versus FAD cells.

## Background

Alzheimer’s disease (AD) is a neurodegenerative disorder and the most common age-related dementia worldwide which has become one of the key unmet medical needs of modern civilization.

AD is clinically and neuropathologically heterogeneous, but two main forms of the disease are described: familial Alzheimer’s disease (FAD), which accounts for approximately 1 % of all affected individuals, and sporadic Alzheimer’s disease (SAD) [1, 2]. FAD has an early onset, before the age of 65 years, and in most cases has a genetic basis—a missense mutation in one of the three genes encoding transmembrane proteins involved in the amyloidogenic pathway: amyloid β precursor protein (AβPP), presenilin 1 (PS1), and presenilin 2 (PS2). Both sporadic and familial AD forms share main neuropathological characteristics, namely β-amyloid plaques, neurofibrillary tangles, and regionalized neuronal loss. However, our understanding of the role that these features of AD play in the etiology of these two forms of the disease remains incomplete and is disputed [3]. The possibility of distinguishing FAD from SAD is of fundamental relevance for the identification of future disease markers, novel drug targets, and therapeutic strategies toward AD. Currently, a very limited number of experimental studies have directly compared FAD and SAD mechanisms.

Most FAD mutations occur in PS1; so far over 227 have been identified in PS1 emphasizing a key role of PS1 in FAD pathogenesis [4]. PS1 is a 48-kDa protein which sustains enzymatic core of the γ-secretase complex responsible for the generation of toxic Aβ from AβPP [5, 6]. These findings strongly support the amyloid cascade hypothesis in the pathogenesis of AD [7, 8]. Nevertheless, recent data indicate differences in SAD and FAD pathogenesis; SAD and FAD present different clinicopathological features [9]. Moreover, in the context of the so-called “cell cycle hypothesis”, we previously reported that peripheral cells from either FAD or SAD patients exhibited distinct cell cycle alterations [10]. These cell cycle disturbances are considered to be systemic manifestations of the proposed aberrant cell cycle activation in differentiated neurons in AD [11–13]. In particular, we reported that SAD compared to FAD B-lymphocytes showed differences in the expression profiles of 90 cell cycle-related genes, and a marked increase in the level of the p21/^CIP1/WAF1^ protein, which promotes G1-arrest [10]. p21 is a cyclin-dependent kinase inhibitor, which inhibits cell cycle progression [14, 15]. However, p21 has been shown to play additional roles depending on cell status as well as its subcellular distribution [14, 16–18]. Notably, p21 can function as a modulator of apoptosis [18–21]. On these grounds, we hypothesized that differences in p21 levels between SAD and FAD lymphocytes could play a role in the cellular response to 2-deoxy-D-ribose (2dRib) that induces apoptosis via a mechanism that involves oxidative stress [22]. Thus, the aim of this study was to compare the apoptotic response of FAD and SAD lymphocytes to 2dRib, and to investigate the role of p21 levels in this response.

## Results

### Higher Survival and Lower Apoptotic Response of FAD Lymphocytes Compared to SAD and Control Cells

**Fig. 1:**
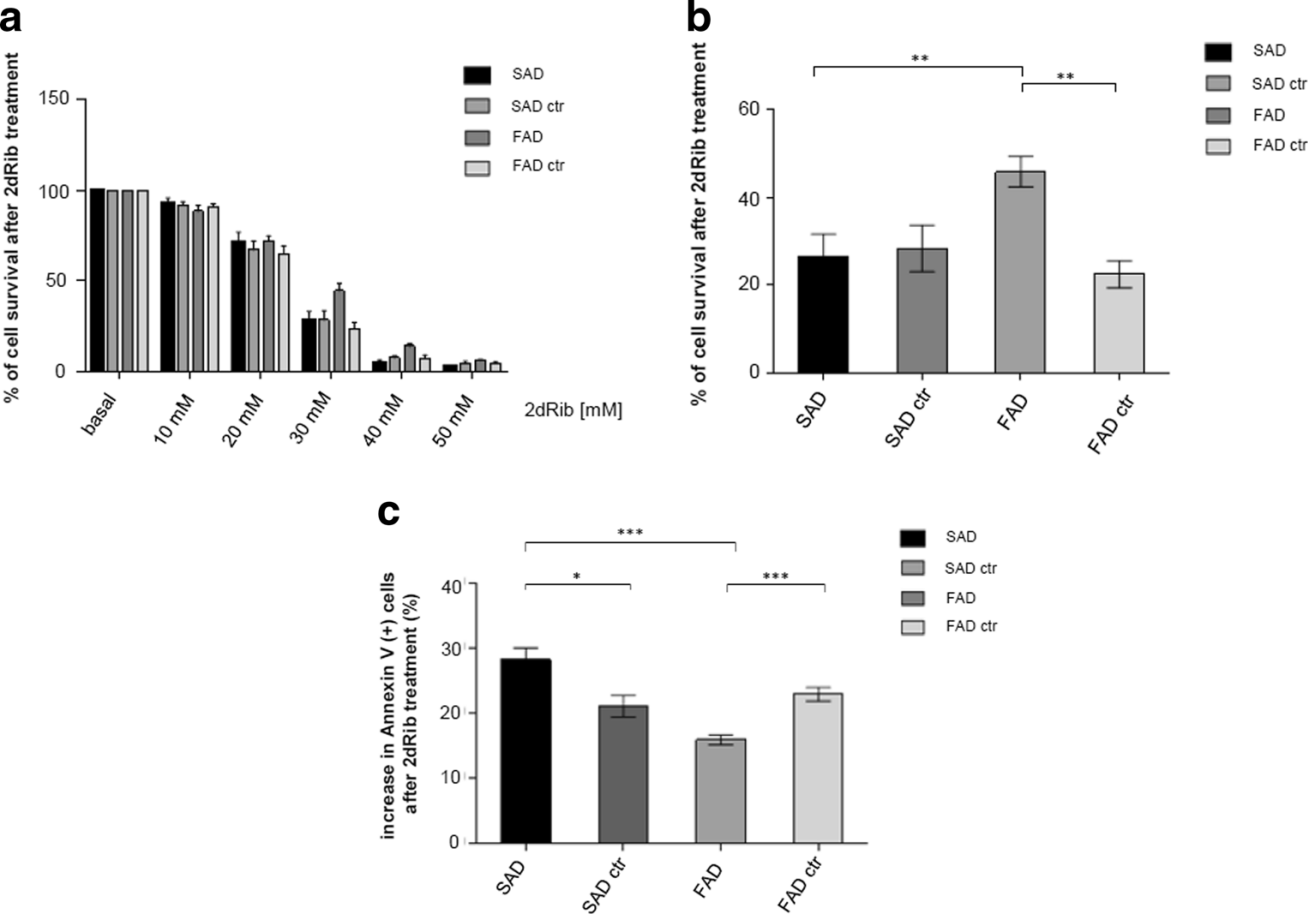
Survival and apoptosis rates of FAD and SAD lymphocytes treated with 2dRib. Lymphocytes from eight SAD patients, eight control individuals age-matched to SAD subjects, seven FAD patients, and eight control individuals age-matched to FAD subjects were analyzed. **a** Cell survival rates measured by MTT assay after seeding of cells at initial density of 1 × 10^6^ × mL^−1^ and incubation for 24 h in the presence of 10, 20, 30, 40, and 50 mM 2dRib. **b** Cell survival rates measured by MTT assay after seeding of cells at initial density of 1 × 10^6^ × mL^−1^ and incubation for 24 h with 30 mM 2dRib. These conditions were used in all further experiments with 2dRib. **c** Apoptosis levels assessed by flow cytometry using Annexin V-FITC and PI in cells incubated for 24 h in the presence of 30 mM 2dRib. Increase in the percentage of apoptotic cells was calculated as percent of cells Annexin V positive after 2dRib stimulation minus percent of Annexin V positive cells without stimulation. Data shown are the means ± standard errors of the means (SEM) calculated using the Mann–Whitney test for at least three independent experiments carried out in each cell line derived from the study subjects. Statistically significant differences are marked: *p* < 0.001, **p < 0.01, *p < 0.05

To determine the dose-response effects of 2dRib, lymphocytes from familial (FAD) and sporadic (SAD) Alzheimer’s patients, as well as age-matched controls were incubated in the absence or in the presence of escalating concentrations (10–50 mM) of 2dRib, and cell viability was determined by MTT assay. As shown in Fig. 1a, 2dRib decreased cell viability in a dose-dependent manner. Further experiments were carried out at 30-mM concentration of 2dRib. Figure 1b shows a significant resistance to 2dRib-induced cell death of lymphocytes from FAD patients, compared to either age-matched controls or lymphocytes from SAD patients. Moreover, no differences were detected between lymphocytes from younger control individuals (FAD ctr) or older (SAD ctr) patients (Fig. 1b), showing that sensitivity to 2dRib is not age-dependent.

Data in Fig. 1c demonstrates that the differences in cell survival between SAD and FAD cells upon 2dRib treatment were reflected in the apoptotic levels measured by flow cytometric analysis of the phosphatidylserine exposure using Annexin V-FITC and Propidium Iodide (PI). The analysis revealed the lower apoptotic response of FAD lymphocytes to 2dRib, compared to SAD cells and both control groups. In addition, it is shown that while SAD cells are more vulnerable to apoptosis than age-matched control cells, the opposite occurs with FAD lymphocytes—they are more resistant to 2dRib-induced apoptosis than their respective control (Fig. 1c).

We sought to further determine the molecular mechanism involved in the apoptotic cell response to 2dRib. Considering that the intrinsic apoptotic pathway is characterized by several morphological and biochemical features that involve mitochondrial membrane depolarization at the early apoptosis stage and lead in the executor stage to nuclear DNA fragmentation, the 2dRib-induced apoptotic response was next assessed by flow cytometry analysis of mitochondrial membrane potential and cellular DNA content.

**Fig. 2:**
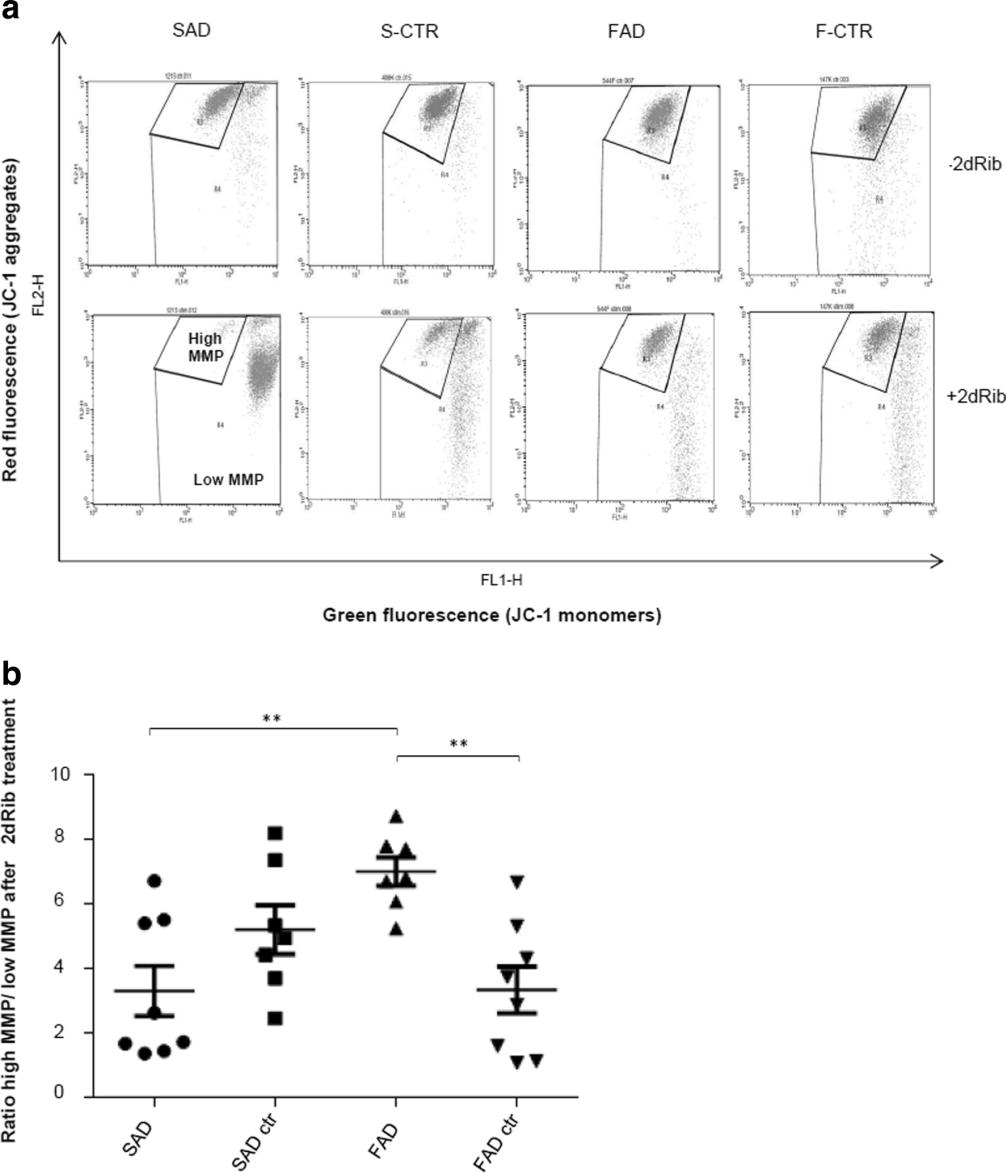
Mitochondrial membrane potential in SAD and FAD lymphocytes treated with 2dRib. Lymphocytes from eight SAD patients, eight control individuals age-matched to SAD subjects, seven FAD patients, and eight control subjects age-matched to FAD subjects were incubated for 24 h in the absence or in the presence of 30 mM 2dRib. After 24 h, cells were stained with cationic dye JC-1 and analyzed by flow cytometry. **a** Representative dot plots showing percentage of cells with high and low MMP in the absence or in the presence of 2dRib. **b** Summary plot showing mean ratios of the percentages of cells with high MMP/low MMP. For each cell line, means ± SEM were calculated based on at least three independent experiments using Mann–Whitney test. Statistically significant differences are marked: ***p < 0.001; **p < 0.01; *p < 0.05

For determining whether the response to 2dRib treatment corresponds to the disruption of active mitochondria, we employed the membrane-permeant JC-1 dye, used in apoptosis studies to monitor alterations in mitochondrial membrane potential (MMP) [23, 24]. At high MMP, JC-1 forms aggregates in mitochondria yielding a red colored emission, while at low MMP, the JC-1 dye is predominantly a monomer that yields green fluorescence. Consequently, mitochondria depolarization is indicated by a change in JC-1 fluorescence from red to green and by a decrease in the red/green fluorescence intensity ratio. As shown in Fig. 2a, b, after 2dRib treatment lymphocytes from SAD patients showed a much higher increase in the percentage of cells with depolarized mitochondrial membranes and a significantly lower mean red/green fluorescence ratio, compared both to age-matched control cells and FAD cells. In contrast, FAD cells demonstrated an increased resistance to mitochondrial membrane depolarization, in agreement with lower apoptosis and a higher survival of FAD cells shown in Fig. 1. Thus, 2dRib strongly induces intrinsic apoptotic pathways involving mitochondria in SAD cells, but FAD cells exhibit protection.

**Fig. 3:**
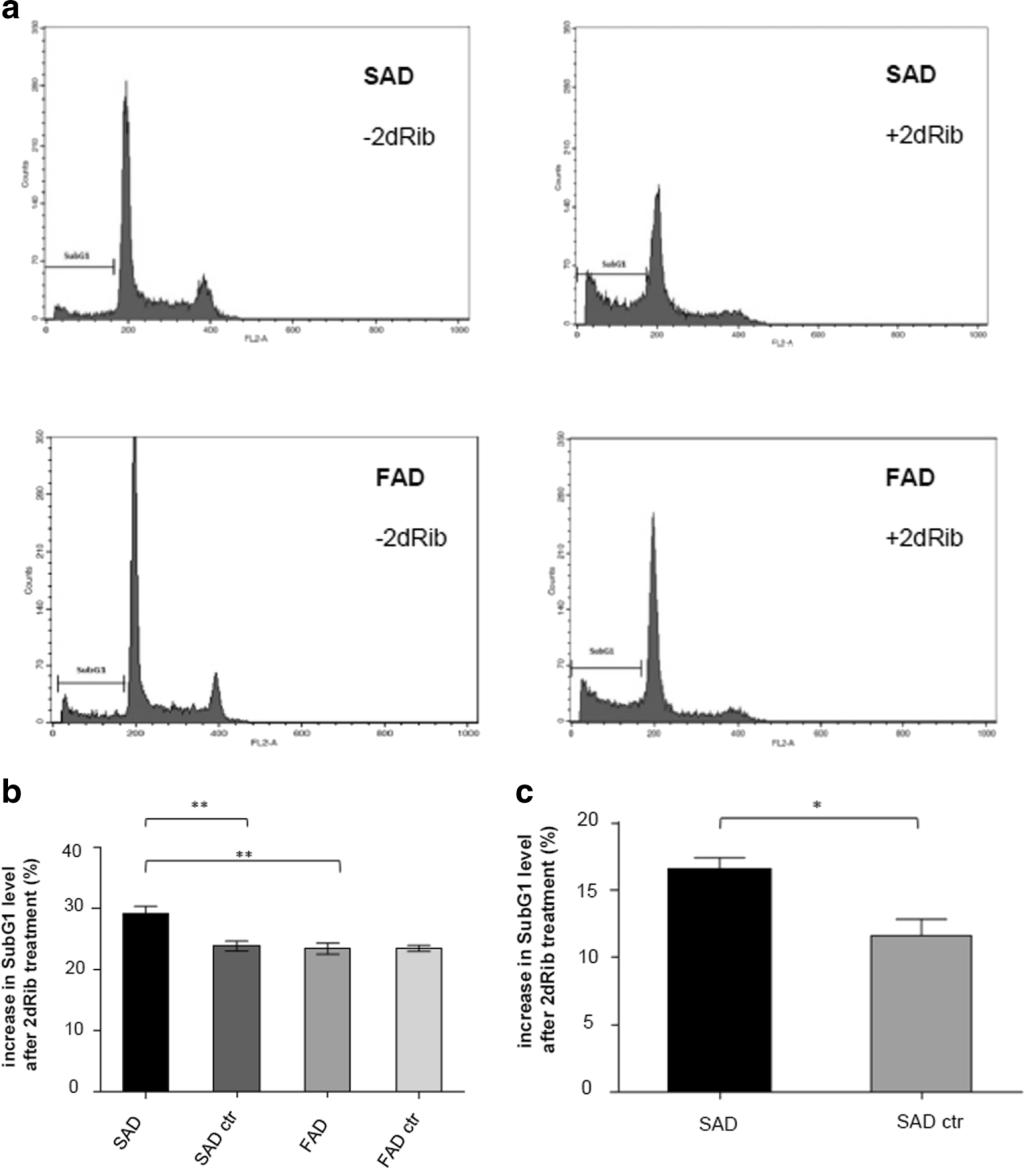
Hypodiploid nuclei content in SAD and FAD lymphocytes treated with 2dRib. Lymphocytes from eight SAD patients, eight control individuals age-matched to SAD subjects, seven FAD patients, and eight control individuals age-matched to FAD subjects were incubated for 24 h in the absence or in the presence of 30 mM 2dRib, permeabilized and stained with PI and analyzed by flow cytometry. Data shown are the mean ± SEM for at least three independent experiments carried out for each of the cell lines. **a** Representative cytograms showing cell cycle profiles based on PI staining. SubG1 phase containing hypodiploid nuclei is marked. **b** Mean increases in SubG1 levels calculated as percentage of 2dRib treated cells gated in SubG1 phase minus percentage of untreated cells gated in SubG1 phase. **c** Mean increases in SubG1 levels calculated as in **b** in B lymphocytes freshly isolated from SAD patients and control subjects after treatment with 10 mM 2dRib. Statistically significant differences (Mann–Whitney test) are marked: **p < 0.01; *p < 0.05

Figure 3a shows a representative experiment of cell cycle status before and after treatment with 2dRib, while Fig. 2b below shows the percentage of cells in subG1 containing hypodiploid nuclei in lymphocytes from controls and AD patients. As demonstrated in Fig. 3a, b, upon 2dRib SAD cells showed a higher accumulation of hypodiploid nuclei compared to cells of age-matched controls and FAD patients, in consistency with results shown in Figs. 1 and 2.

Figure 3c demonstrates, for comparative purposes, the effect of 2dRib treatment in freshly isolated B-lymphocytes from control and SAD subjects. It is noteworthy that a higher than in controls percentage of cells in subG1 after the 2dRib addition in SAD lymphocytes freshly isolated from patients’ blood corresponds to the same result in SAD immortalized lymphocytes (Fig. 3c). This data is in agreement with previous reports indicating that the cellular response of lymphocytes from AD patients is not affected by the viral transformation [25, 26].

Altogether, this data indicated that SAD lymphocytes are more vulnerable to apoptosis evoked by redox stress induced by 2dRib, than cells from FAD patients, and controls age-matched to SAD and FAD subjects. In addition, our results suggest activation of the intrinsic mitochondrial activated pathway by 2dRib in SAD cells resulting in DNA fragmentation and the resistance of FAD cells to 2dRib-induced mitochondrial membrane depolarization and intrinsic apoptosis.

### Molecular Mechanism Protecting FAD Cells from 2dRib-Induced Apoptosis

#### Increased Protein and mRNA Levels of p21 in FAD Cells after 2dRib Treatment

**Fig. 4:**
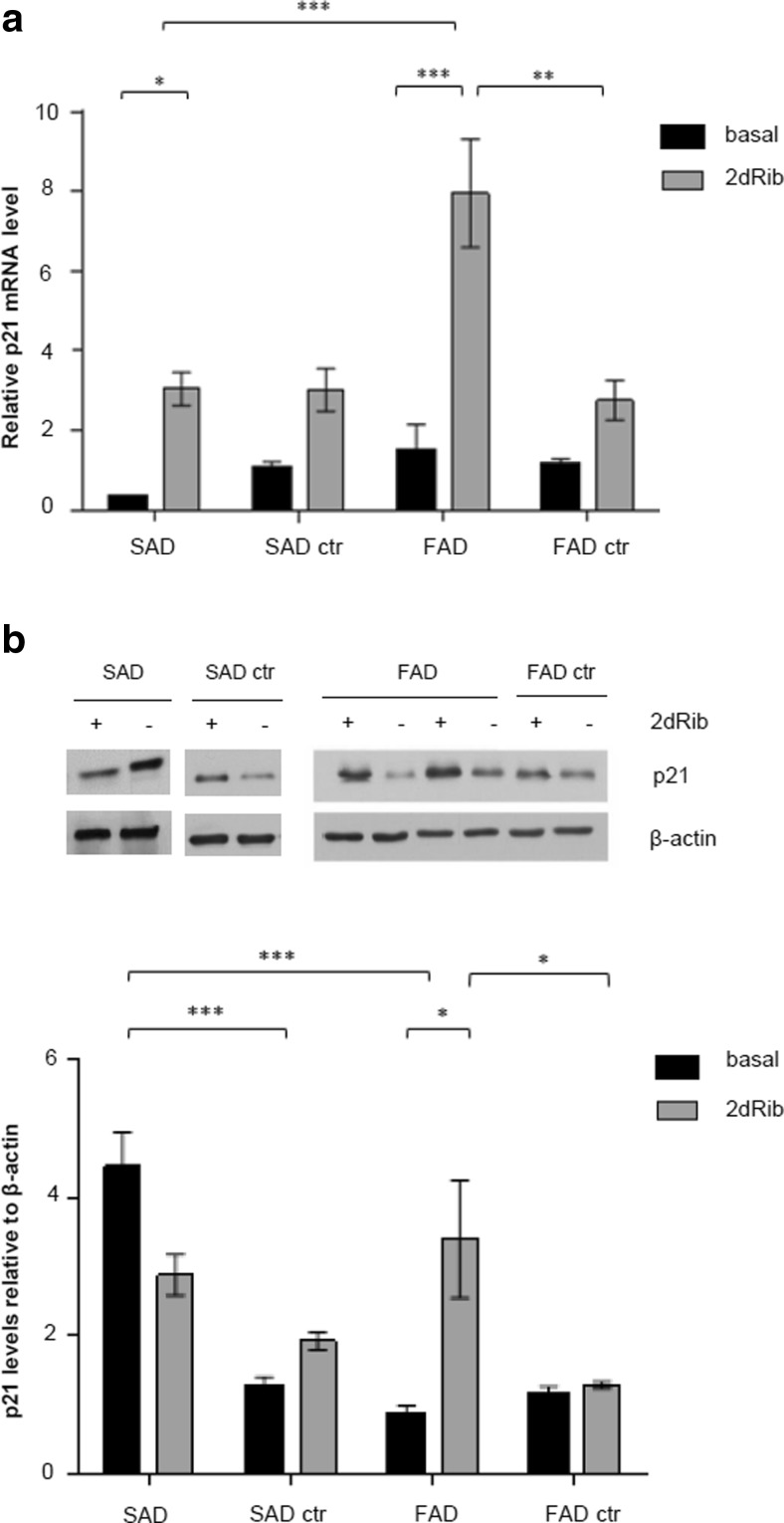
p21 mRNA and protein levels in SAD and FAD lymphocytes before and after 2dRib treatment. Lymphocytes from eight SAD patients, eight SAD age-matched control subjects, five FAD patients, and five FAD age-matched control subjects were incubated for 24 h in the absence or in the presence of 30 mM 2dRib, and analyzed. **a** Relative p21 messenger RNA (mRNA) abundance in lymphocytes. Isolated total mRNA was subjected to quantitative real-time polymerase chain reaction. Relative p21 mRNA levels were normalized to β-actin expression, and values for control cells were set as 1. **b** p21 protein levels in lymphocytes were assessed with immunoblotting (representative immunoblots are shown on *top*), and analyzed using densitometry (*below*); p21 protein levels were quantified in relation to β-actin. All quantitative data represents the means ± SEM for at least three independent experiments carried out for each cell line. Statistically significant differences (two-way ANOVA test with Tukey post-test) are marked ***p < 0.001, *p < 0.05

We previously reported significant differences in p21 basal levels in SAD lymphocytes compared to cells from control or FAD individuals. Since it is known that p21, besides controlling the G1/S checkpoint, can regulate apoptosis [27, 28], we were interested in studying whether p21 levels play a role in the cellular response of AD cells to 2dRib treatment. The mRNA and protein p21 levels in SAD and FAD cells compared to age-matched control cells are shown in Fig. 4. In agreement with our previous work [10], we detected higher basal levels of the 21 protein in lymphocytes from SAD cells compared to either cells from FAD or control individuals (Fig. 4b). It was considered that the increase in p21 levels promoted G1 arrest, and therefore SAD cells showed a prolonged G1 phase [10]. Interestingly, the basal levels of p21 mRNA levels in SAD cells did not increase (Fig. 4a), suggesting that the increased basal p21 levels in SAD cells result from a deficient degradation of the p21 protein in the proteasome.

Despite the fact that FAD cells show a lower basal p21 cellular content than SAD cells, particularly high increases in p21 mRNA and protein levels occurred in FAD cells in response to 2dRib (Fig. 4).

#### Intracellular Localization of p21. Increased p21 Protein Levels in Cytoplasm of FAD Cells After Apoptotic Stimulation

**Fig. 5:**
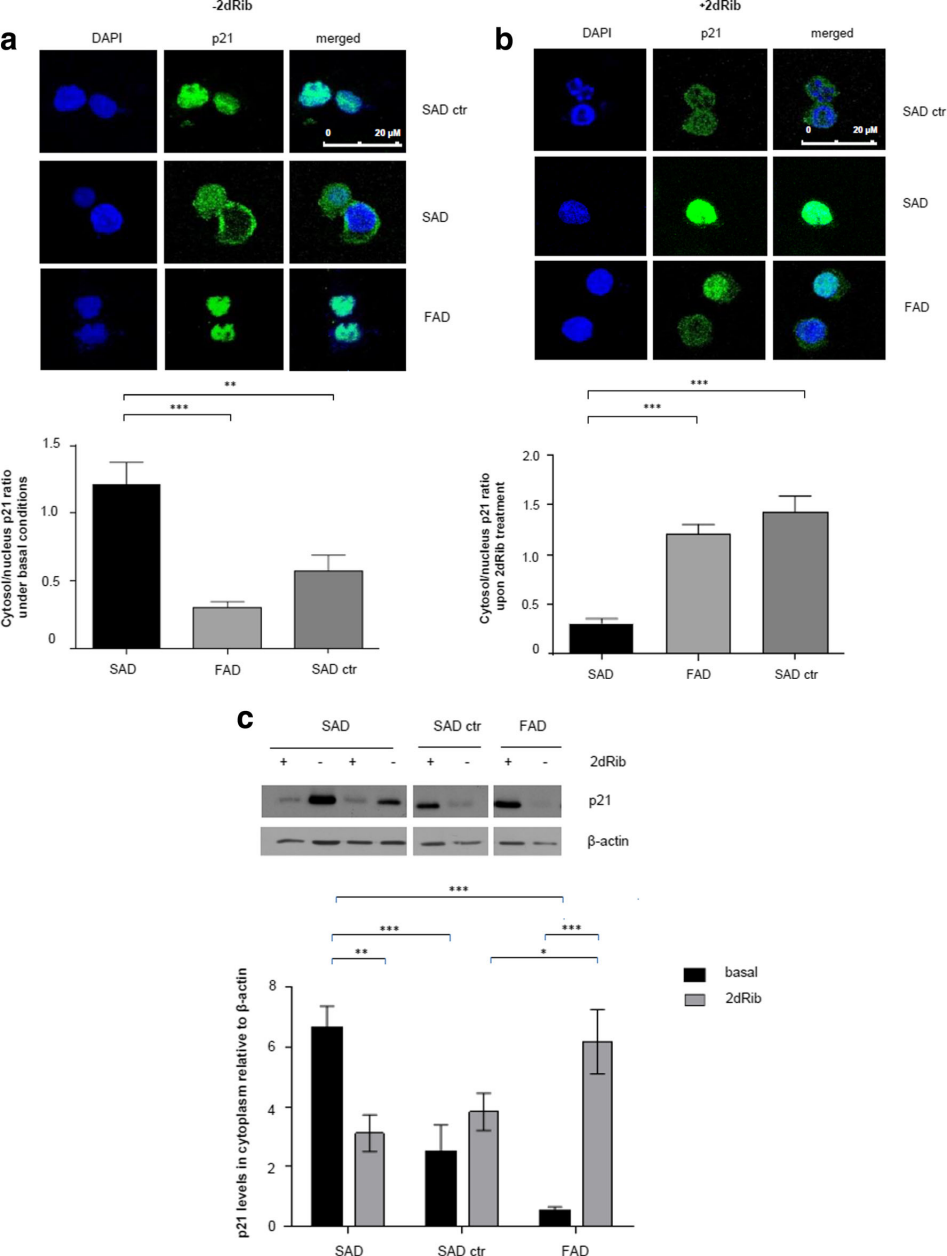
Subcellular localization of p21 in SAD and FAD lymphocytes before and after 2dRib treatment. Subcellular localization of p21 was assessed by confocal scanning microscopy (**a**) and (**b**), and by immunoblotting (**c**). Lymphocytes were incubated for 24 h in the absence (**a**) or in the presence (**b**) of 30 mM 2dRib. **a, b** Cells were collected and stained with anti-p21 antibody followed by secondary antibody labeled with Alexa Fluor 488. 4′,6-Diamidino-2-phenylindole (DAPI) was used for nuclear staining. Confocal images are merged, magnification ×63. Quantitative analysis of p21 cytosol/nucleus ratio was obtained using ImageJ software. Shown are means ± SEM from representative experiments in which at least 10 cells were analyzed (**p < 0.01, ***p < 0.001; Mann–Whitney test). **c** Immunoblotting of p21 protein in cytosolic fraction of lymphocytes from six SAD patients, six control subjects age-matched to SAD patients, and from six FAD patients. Representative immunoblots are shown on *top*, the densitometric quantitation of all immunoblots are shown *below*. Data represents the means ± SEM for at least three independent experiments carried out for each cell line. Statistically significant differences (two-way ANOVA test) are marked ***p < 0.001, **p < 0.01, *p < 0.05

p21 has been considered a protein showing different nuclear versus cytosolic functions [29, 30]. Therefore, we decided to examine whether there are differences in the subcellular localization of p21 in control, SAD, and FAD lymphocytes under basal conditions and after 2dRib stimulation. For this purpose, cells were immunostained with an anti-p21 antibody and processed for confocal laser imaging. In addition, we performed cell fractionation and analyzed the protein extracts by immunoblotting. Figure 5a shows marked differences in the subcellular distribution of p21 between SAD and FAD cells under basal conditions. It demonstrates that p21 is located preferentially in the nucleus of FAD and control cells, while it appears that in SAD cells it is distributed prevailingly in the cytoplasm. Similar results were obtained by immunoblotting, as shown in Fig. 5c.

After 2dRib treatment, the analysis of p21 subcellular distribution, either by confocal microscopy (Fig. 5b) or by immunoblotting (Fig. 5c) revealed an opposite response to 2dRib in SAD cells compared to control and FAD cells. Cytosolic p21 levels increased in control and most significantly in FAD cells, while they decreased in SAD cells (Fig. 5b, c). In Fig. 5b, DAPI images after treatment with 30 mM 2dRib depict fragmented, apoptotic nuclei in SAD and control cells. In contrast, in FAD cells nuclei are without such signs, which is associated with a predominantly cytosolic localization of p21 and a higher level of p21 than in SAD and control cells.

#### p53-Dependent Transcriptional Regulation of p21 in FAD Cells

**Fig. 6:**
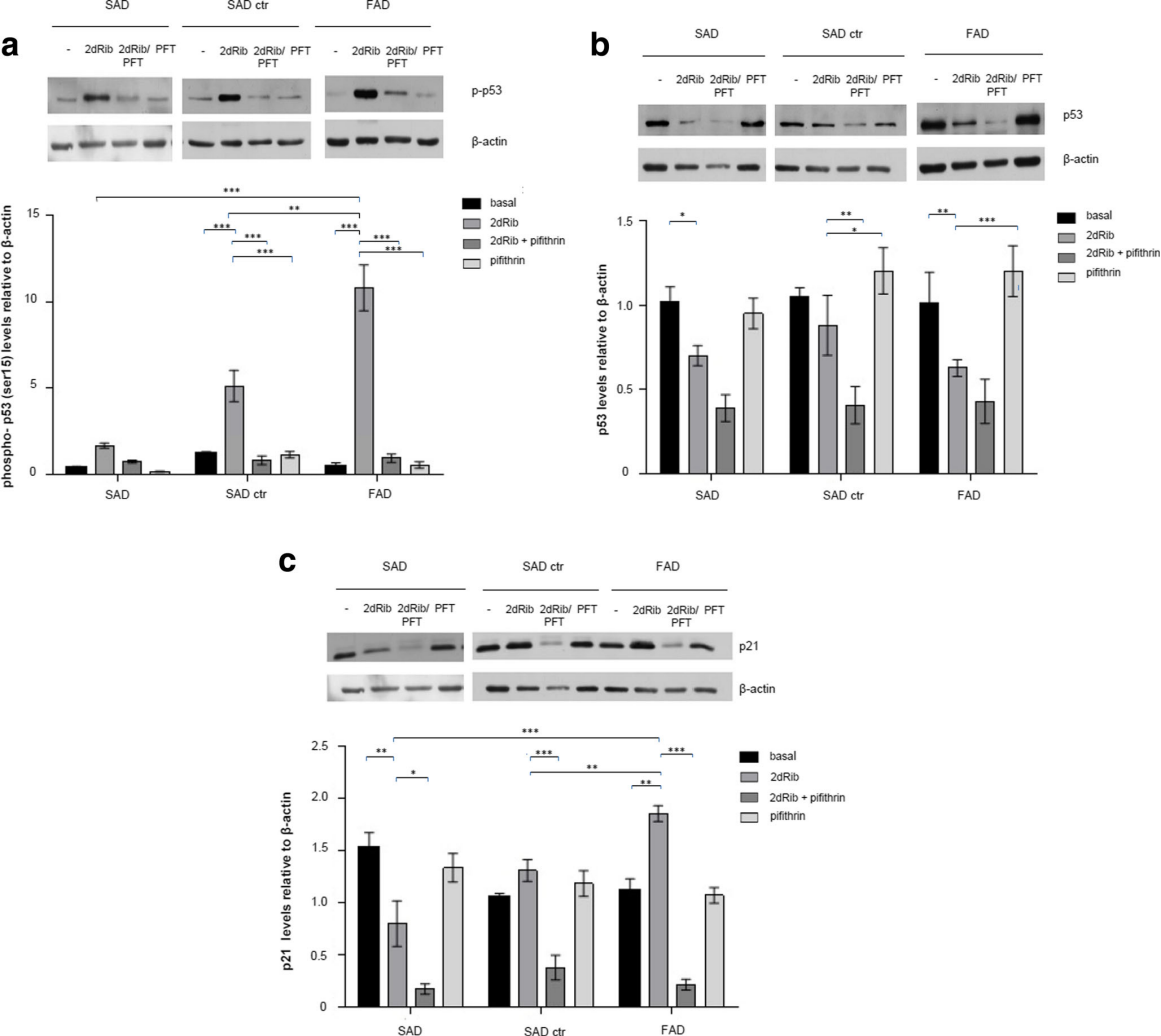
Effects of 2dRib and PFT-α on cellular levels of phospho-p53 (**a**), total p53 (**b**), and p21 (**c**) in FAD and SAD lymphocytes. Cells from seven SAD patients, seven control subjects and from FAD patients were incubated for 24 h in the absence or in the presence of 30 mM 2dRib and/or 30 μM PFT-α and analyzed by immunoblotting. Protein levels were quantified by densitometry in relation to β-actin and are shown below the representative immunoblots. The densitometric data represents the means ± SEM for at least three independent experiments carried out for each cell line. Statistically significant differences (two-way ANOVA test with Tukey post-test) are marked ***p < 0.001; **p < 0.01; *p < 0.05

**Fig. 7:**
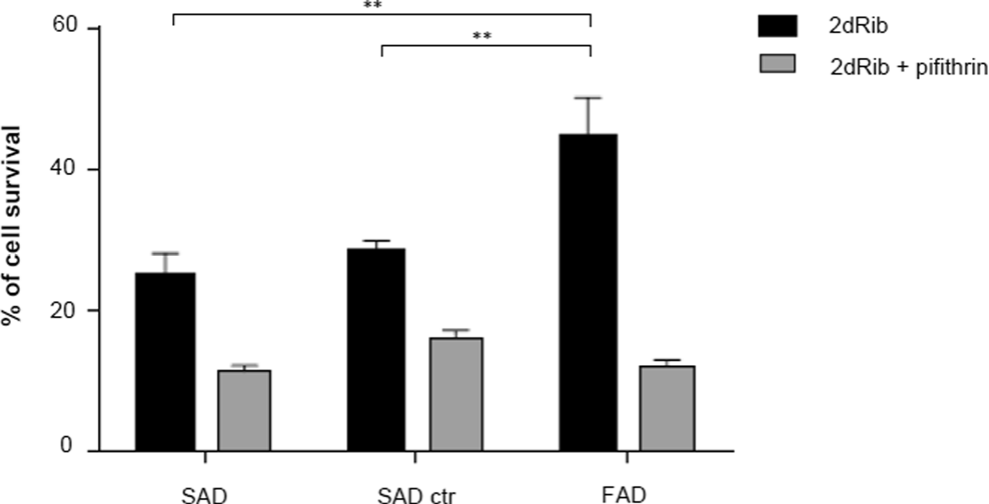
Effect of PFT-α and 2dRib treatment on cell survival. Lymphocytes from seven SAD patients, seven control subjects and from FAD patients were incubated for 24 h in the absence or in the presence of 30 mM 2dRib and 30 μM PFT-α. Cell survival was assessed by the MTT assay. Results represent the percent of cell survival of treated cells referred to untreated ones. Data shown are the means ± SEM for at least three independent experiments carried out for each cell line. Statistically significant differences (two-way ANOVA test with Tukey post-test) are marked **p < 0.01; *p < 0.05

As transcriptional activation of the p21 gene can occur through p53-dependent and p53-independent mechanisms, we first tested whether transcriptional activation of the p21 gene was dependent on p53 activity. For this purpose, we determined by immunoblotting the phosphorylation of p53 at Ser 15, together with total p53 and p21 levels in the absence and in the presence of the p53 inhibitor PFT-α [31], (Fig. 6), and analyzed the influence of this inhibitor on cell survival after 2dRib treatment (Fig. 7).

As shown in Fig. 6a, the addition of 2dRib appears to activate the p53 pathway in all cell types, as reflected by the increase in the phosphorylated (active) form of the protein, although total p53 levels rather decreased (Fig. 6b). The highest increase in the level of the active p53 form occurred in FAD cells, which corresponds to the highest increase in p21 level in FAD cells compared to control and SAD cells (Fig. 6c). On the other hand, the addition of PFT-α effectively decreased the 2dRib-induced increase in Ser15 p53 levels, and reduced the cellular content of p21, while PFT-α alone did not affect the basal levels of p53 and p21. Together, these results suggest that 2dRib activates the transcription of p21 in all cell types, but it seems that FAD cells exhibit an enhanced response to the 2dRib-induced increase in p21 levels. Finally, we assessed the effects of PFT-α on 2dRib-induced cell responses. PFT-α did not affect the survival of control or AD cells, as assessed by the MTT assay in the absence of 2dRib (not shown), but increased cell death induced by 2dRib in all cell types (Fig. 7). Under these conditions, death of FAD lymphocytes is the same than that of control or SAD cells. Altogether, this data suggests that the higher survival and lower apoptotic response to 2dRib in FAD lymphocytes compared to SAD and control cells is determined by the highest activation of the p53-p21 pathway and the shift of the p21 localization from the nucleus to the cytoplasm.

## Discussion

This work was undertaken to study whether the reported differences in p21 levels between lymphocytes from SAD and FAD individuals carrying seven different PS1 mutations (M139V, L153V, H163R, S170F, F177L, I213F, E318G) [10], play a role in cell fate (survival/death) when they are challenged by oxidative stress, like the one provided by the reducing sugar 2dRib [32, 33]. We report here that peripheral B lymphocytes from SAD and FAD patients differ in cellular response to this apoptotic stimulus. Cells from PS1 mutation carriers are more resistant to 2dRib-induced cell death when compared with age-matched controls or SAD patients. To the best of our knowledge, this is the first report comparing the vulnerability of SAD and FAD cells against acute oxidative stress.

Increasing evidence indicates that SAD and FAD patients present distinct clinical and pathological features [9]. Moreover, several early pathogenic processes seem to differentiate SAD from FAD, including different genetic and environmental factors (reviewed in [34, 35]). For example, SAD risk genes identified by genome-wide association studies comprise multiple loci associated with lipid metabolism, innate immunity and cell signaling, but not APP, PS1, or PS2 [36, 37]. In addition, hundreds of recent clinical trials with potential drugs targeting the amyloidogenic pathway have failed. One of the postulated reasons is that these therapeutics were based and tested in preclinical studies using FAD models that are not fully applicable to SAD. The identification of molecular differences between SAD and FAD is thus important for further drug development. In addition, there is an urgent need for a blood biochemical test that can support the early diagnosis of AD and all biological modalities involving blood cells and plasma are explored for this aim [38]. From this perspective, knowledge on possible SAD and FAD differences in phenotypes and protein profiles including these of blood cells is of growing significance.

While this is the first comparison of stress response in SAD and FAD cells, selective impairment of cell survival mechanisms in peripheral cells derived from AD patients was previously reported [39, 40], although there is some controversy as to whether AD cells are more resistant or more vulnerable to a number of cell stressors [41, 42].

2dRib is known to induce apoptosis mainly by depleting reduced glutathione (GSH), the cellular thiol responsible for preserving reduced intracellular environment and blocking the accumulation of reactive oxygen species (ROS) [32, 33]. The decrease of cellular GSH results in the ROS-induced activation of mitochondrial apoptotic signaling [43]. In our study, cell death induced by 2dRib treatment showed in all SAD, FAD, and control lymphocytes characteristics of intrinsic apoptosis involving mitochondrial pathway, in agreement with the known cellular mechanism of 2dRib activity. However, we found a lower sensitivity of FAD lymphocytes to 2dRib accompanied by a lower dissipation of the mitochondrial membrane potential, and a decreased percentage of apoptotic cells compared to lymphocytes from SAD or control individuals.

In lymphocytes, the p53 tumor suppressor protein is a main sensor of intrinsic stress stimuli such as oxidizing intracellular environments [44]. The phosphorylation of p53 at Ser15 plays a fundamental function in promoting p53 transcriptional activity including the expression of p21, the key target of p53 (reviewed in [45]). In our study, 2dRib induced the phosphorylation of p53 at Ser15 in all cell types; however, the level of phosphorylated p53 in FAD cells was significantly higher than in control or SAD cells, and correlated with the 3-fold increase in p21 transcripts in FAD compared to control or SAD cells. Thus, the magnitude of the p53-mediated activation of p21 gene transcription was strikingly greater in FAD cells. As p21 is a key regulator of apoptosis (reviewed in [14, 16]), this difference appears to account for the distinct apoptotic response of FAD to 2dRib.

Interestingly, despite transcription activation, levels of the p21 protein either decreased in SAD cells or did not change in control lymphocytes. It is of note, and in accordance to our previous reports [10], that SAD cells showed higher basal levels of the p21 protein than FAD or control lymphocytes. Taken together, these results suggest differences in the rates of p21 transcription and degradation between control, SAD, and FAD lymphocytes.

In addition to the increased transcriptional activation of the p21 gene and the increased cellular content of p21 observed in FAD lymphocytes after 2dRib treatment, we also found an increase in the p21 cytosolic content of FAD cells. This result explains the lower apoptotic response of FAD lymphocytes, because p21 is associated with cell cycle arrest when present in the nucleus, and with the apoptotic block when localized in the cytoplasm (reviewed in [46, 47]). For example, cytoplasmic p21 has been reported to protect etoposide-induced apoptosis in leukemia cells [28], and it is thought to be a positive modulator of cell survival [30, 48]. Moreover, cytoplasmically localized p21 binds to and inhibits the activity of proteins directly involved in the induction of apoptosis, including procaspase 3, caspase 8, caspase 10, stress-activated protein kinases (SAPKs) and apoptosis signal-regulating kinase 1 (ASK1) [18, 19, 49, 50].

Together, our results suggest that the increased activation of p21 mRNA expression in a p53-dependent manner, together with a preferential cytosolic localization of p21 and apparently higher p21 protein stability, confers a survival advantage to FAD lymphocytes compared with control or SAD cells.

Importantly, B lymphocytes contain high levels of PS1 [51]. Thus, our data suggest substantially altered functions of mutPS1 versus wtPS1 in the oxidative stress response which involves the activation of the p53–p21 signaling pathway. Under basal conditions, FAD PS1 mutants are known to contribute to the endoplasmic reticulum (ER) stress, what in turn exaggerates Ca^2+^ release from the ER and leads to increased mitochondrial dysfunctions, as demonstrated in human FAD brain and lymphocytes, and in FAD murine models [52–55]. Our study indicates that under acute oxidative stress evoked by 2dRib, PS1 mutants strongly exacerbate phosphorylation of p53 thus exhibiting gain of function effect over wtPS1. Moreover, in the presence of mutPS1, p53-dependent enhancement of p21 protein levels seems to be associated with retention of p21 in the cytoplasm, where p21 plays anti-apoptotic function. These activities associated with mutPS1 probably represent a compensatory mechanism against acute oxidative stress, preventing depolarization of mitochondrial membrane and apoptosis in FAD cells, and resulting in higher survival. In SAD cells expressing wtPS1, this mechanism is absent. As SAD cells like control cells express wild-type PS1 (wtPS1), the observed higher apoptotic response in SAD versus control cells most likely reflects the pathological versus physiological intracellular environment.

Existing data on the cross-talk between PS1 and p53 in an apoptotic context is only fragmentary. PS1 is an enzymatic core of a γ-secretase complex which participates in the generation of Aβ peptides from its precursor AβPP [2, 56]. The direct mechanistic link between PS1 and p53 was the demonstration that APP-derived amyloid intracellular C-terminal domain (AICD) fragments generated by γ-secretase can function as transcription factors activating p53 transcription, and that some PS1 mutants augment this process, resulting in higher levels of the p53 protein [57, 58]. Moreover, upregulated p53 levels were shown to be associated with a decreased proteasome activity in cells with mutated PS1 [59]. Consistently with low APP expression levels in B cells [60], p53 transcriptional regulation was not detected in our study in FAD cells; neither was lower degradation. Instead, this is the first documentation of the link between PS1 mutants and the activation of p53 by phosphorylation at Ser15, resulting in the upregulation of p21 levels. Moreover, this is the first description of the association of PS1 mutants with the mechanisms responsible for cytoplasmic p21 localization, known to determine anti-apoptotic cellular response.

At present, we can only speculate about the molecular mechanisms involved in the effects of PS1 mutations controlling the cellular response to 2dRib, and several hypothesis can be formed. In addition to APP, there are over 90 γ-secretase substrates including Notch, and γ-secretase-dependent effects on apoptosis are currently explored [61]. Moreover, PS1 plays a number of γ-secretase-independent functions in processes associated with cell fate and apoptosis regulation, such as Wnt/β-catenin signaling, mitochondrial proapoptotic PS1-associated protein (PSAP) signaling, and calcium homeostasis (reviewed in [62]). Further studies are required for identification of signaling pathways leading from mutPS1 to p53 phosphorylation.

Mutations in PS1 have been associated with an increase in the vulnerability of neurons to several damaging factors [63, 64] in contrast to the apoptosis resistance detected in lymphocytes from FAD patients. However, contradictory data showing lower apoptosis in neurons bearing mutPS1 also exists (reviewed in [35]). In this regard, it is worth highlighting that FAD mutations in PS1 were shown to have distinct features depending on the location in the PS1 structure, especially differing location in the PS1 first cytoplasmic loop and transmembrane regions [65–67]. To minimize this source of variability, in this study, all investigated mutations were located outside the first loop, and six of them in the transmembrane regions (Table 2). Importantly, our previous report marked the differences in p21 content and cell cycle regulation between SAD and FAD lymphocytes bearing the same PS1 mutations as analyzed here [10]. It appears, therefore, that in FAD cells, there is a precise modulation of the total levels of p21 both in basal conditions [10] and upon oxidative stress (this manuscript), leading to G1/S progression and increasing the total cellular and cytoplasmic p21 protein content, and an increased resistance to 2dRib-induced apoptosis.

Neuronal cell cycle dysfunctions that lead to apoptosis are believed to contribute to AD pathogenesis [11, 68, 69]. Nevertheless, one has to take into account that although the dysfunctions of cell cycle control in the brain and in lymphocytes may have similar causes, the mitogenic stimulation to enter the cell cycle has different consequences. Lymphocytes from FAD patients show a shorter G1 phase and an increased resistance to 2dRib-induced cell death than cells derived from non-demented individuals or SAD patients. These features might represent an adaptive response for FAD cells that are exposed to accumulating oxidative challenges and degenerative processes during disease progression. It has been considered that susceptible neurons in AD survive for a long time in a compromised way by delaying the apoptotic process, a mechanism termed abortive apoptosis [70]. However, cell cycle re-activation in already adult neurons results in cellular dysfunction, premature cell death, and thus neurodegeneration [8, 12, 71].

Although the observations made in FAD lymphocytes may not exactly reflect the changes occurring in FAD brains, the fact that p21 levels change in response to both mitogenic stimuli and cellular stressors, may offer an explanation for the relationship between cellular stress and unscheduled cell cycle entry observed in susceptible AD neurons in agreement with the “two-hit” hypothesis [12, 72, 73].

In summary, we have detected important differences between B lymphocytes from SAD and FAD patients when it comes to the mechanisms involved in regulation of p53 activity, cellular p21 levels and cell fate in response to an oxidative challenge. FAD PS1 mutations proved to be associated with the p53-mediated increase in p21 transcription and cytoplasmic localization, resulting in a survival advantage against 2dRib-induced apoptosis. This compensatory mechanism is absent in SAD cells bearing wtPS1. Thus, caution should be taken in extrapolating data obtained from cellular or animal models based in FAD mutations, as they may not be relevant in SAD. Consistently, therapeutic designs should take into account the possible effect variability in SAD versus FAD cells. Particularly, the possible differential responses of FAD versus SAD B-lymphocytes are important for active vaccination strategies in AD. This data is also relevant for the recently developing area of studies concerning the role of systemic immune cells in AD pathogenesis and for the development of new blood-based diagnostic methodologies targeting proteins and genes in lymphocytes.

## Materials and Methods

### Subjects

Demographics and genetic characteristics of all subjects enrolled in this study are provided in Tables 1 and 2. All individuals were enrolled in the Department of Neurology at the Central Clinical Hospital (MSWiA) in Warsaw, Poland, or in the Hospital Doce de Octubre in Madrid, Spain. A clinical diagnosis of probable AD was performed according to the criteria of the Diagnostic and Statistical Manual of Mental Disorders, 4th edition (DSM-IV) and the criteria of the National Institute on Aging and Alzheimer’s Association workgroups [74]. All subjects were examined by a neurologist and a neuropsychologist. The diagnosis was based on an interview, an objective and neurological examination, a cognitive evaluation, and laboratory and radiological tests; a computer tomography scan with an assessment of hippocampal fissure was obtained for each patient. The laboratory tests diagnosing FAD included screening of PS1 gene either using RT-PCR method or genomic DNA analysis, as described earlier [75, 76]. The cognitive status was quantified using the Mini-Mental State Examination (MMSE). Seven subjects were diagnosed as FAD, each with at least one first-degree relative who had suffered from early onset, AD-type dementia, and with a mutation in the PS1 gene. All control subjects were non-demented, with no clinical signs of neurological or psychiatric diseases, and normal cognitive and neuropsychological test results. The average education level of the control and AD groups was not different, and the proportions of female to male subjects were even. All study protocols were approved both by the Ethics Committee for Studies on Human Subjects at the MSWiA Hospital in Warsaw (agreement nos. 89/2014, 102/2010), Poland, and by the Spanish Council of Higher Research Institutional Review Board, and are in compliance with the National and European Union legislation, and the Code of Ethical Principles for Medical Research Involving Human Subjects of the World Medical Association. Peripheral blood samples were collected from all subjects after obtaining written informed consent from the patients or their representatives. None of the subjects was affected with neoplastic or autoimmune diseases when the blood samples were taken.

**Table 1:** Characteristics of the study subjects

Subjects	Mean age of onset	MMSE
FAD (7 patients)	38.1 ± 7.3	13.4 ± 8.5
F-CTR (12 middle-aged subjects)	38.5 ± 9.6	≥25
SAD (17 patients)	68.6 ± 9.8	14.4 ± 7.3
S-CTR (17 elderly subjects)	72.2 ± 5.9	27.9 ± 1.0

**Table 2:** Characteristic of FAD subjects in the Polish population

Mutation in PS1 and its localization^a^	MMSE	ApoE
PS1 M139 V, TD^a^ II	7	E3/E3
PS1 L153 V, TD II	18	E3/E3
PS1 H163R, TD III	11	E3/E3
PS1 S170F, TD III	11	E3/E3
PS1 F177 L, TD III	21	E3/E3
PS1 I213F, TD IV	0	E3/E4
PS1 E318G, L II	26	E3/E3

^a^Localization of FAD mutations in the PS1 structure is based on nine transmembrane PS1 model [78]

*TD* transmembrane domain, *L* loop

### Cell Lines

Peripheral blood lymphocytes were separated from heparinized blood by centrifugation on Ficoll-Hypaque (Sigma-Aldrich, St. Louis, MO, USA). Cells were washed twice with phosphate buffered saline (PBS), counted, and resuspended at the desired concentration. Immortalized B-lymphocytes were established with the Epstein–Barr virus using the standard procedure as described in [10]. Cells were grown in suspension in T flasks in an upright position, in approximately 10 mL RPMI-1640 medium that contained 2 mM L-glutamine, 100 mg/mL penicillin/streptomycin, and 10 % (*v*/*v*) fetal bovine serum, and were maintained in a humidified 5 % CO_2_ incubator at 37 °C. All reagents needed for cell culture were purchased in Sigma-Aldrich. Fluid was routinely changed every 2 days by removing the medium above the settled cells and replacing it with an equal volume of fresh medium.

### Preparation of Whole-Cell Extracts and Subcellular Fractionation

To prepare whole-cell extracts, cells were harvested, washed in PBS, and then lysed in an ice-cold buffer (50 mMTris pH 7.4, 150 mM NaCl, 50 mM NaF, 1 % Nonidet P-40), containing 1 mM sodium orthovanadate, 1 mM PMSF, 1 mM sodium pyrophosphate, and protease inhibitor Complete Mini Mixture (Roche). To separate the cytosolic and nuclear fractions, cells were harvested, washed in PBS, and then lysed in an ice-cold hypotonic buffer (10 mM HEPES, pH 7.9, 10 mM KCl, 0.1 mM ethylenediaminetetraacetic acid (EDTA), 0.1 mM ethylene glycol tetraacetic acid (EGTA), 1 mM sodium orthovanadate, 1 mM sodium pyrophosphate, 1 mM PMSF, and protease inhibitor mixture). After extraction on ice for 15 min, 0.5 % Nonidet P-40 was added and the lysed cells were centrifuged at 4,000 rpm for 10 min. Supernatants containing cytosolic proteins were separated, and nuclei were washed twice with the hypotonic buffer, and then lysed in a hypertonic buffer (20 mM HEPES, pH 7.9, 0.4 M NaCl, 1 mM EDTA, 1 mM EGTA, 1 mM sodium orthovanadate, 1 mM sodium pyrophosphate, 1 mM PMSF, and protease inhibitor mixture). After extraction on ice for 30 min, the samples were centrifuged at 15,000 rpm for 15 min at 4 °C. Antibodies to α-tubulin and to lamin B were used to assess the purity of the fractions. The protein content of the extracts was determined by the Pierce BCA Protein Assay kit (Thermo Scientific, Waltham, MA, USA).

### Immunoblotting

Samples were separated by sodium dodecyl sulfate-polyacrylamide gel electrophoresis (SDS-PAGE), and transferred to poly(vinylidene) fluoride (PVDF) membrane (Immobilon-P, Bio-Rad Laboratories, Richmond, CA, USA). Equal amounts of protein (20–50 μg) were loaded in each lane. Uniformity of sample loading and the integrity of transfer were verified by staining with Ponceau-S (Sigma-Aldrich). After transfer, the PVDF membranes were blocked with nonfat milk and incubated overnight at 4 °C with primary antibodies, followed by secondary antibodies conjugated to horseradish peroxidase (HRP). Detection of the immunoreaction was performed with an enhanced chemiluminescence (ECL) kit (Amersham BioSciences, Amersham, UK). Protein band densities were quantified using Image J software (NIH, Bethesda, MD, USA) after scanning the images with a GS-800 densitometer from Bio-Rad.

### Antibodies

Anti-p21 rabbit monoclonal antibody (1:1,000), rabbit polyclonal anti-p53 antibody (1:1,000), and phospho-p53 (ser15) (1:1,000) were purchased from Cell Signaling (Danvers, MA, USA). Mouse monoclonal antibodies: anti-β-actin (1:1,000), anti-α-tubulin (1:1,000), and anti-Lamin B (1:1,000) were purchased from Santa Cruz Biotechnology (Santa Cruz, CA, USA).

### Quantitative Real-Time PCR

Lymphocytes from controls, as well as SAD and FAD individuals were seeded at an initial density of 1 × 10^6^ cells × mL^−1^ in RPMI and incubated for 24 h with or without 30 mM 2-deoxy-D-ribose. After stimulation, cells were collected and total RNA was extracted from cell cultures using Trizol™ reagent (Invitrogen, Waltham, MA, USA). RNA yields were quantified spectrophotometrically and RNA quality was checked by the A260/A280 ratio and on a 1.2 % agarose gel to observe the integrity of 18S and 28S rRNA. RNA was then treated with DNase I Amplification Grade (Invitrogen). One microgram was reverse transcribed with the Superscript III Reverse Transcriptase kit (Invitrogen). Quantitative real-time PCR was performed in triplicates using TaqMan™ Universal PCR MasterMix No Amperase UNG (Applied Biosystems, Carlsbad, CA, USA) reagent according to the manufacturer’s protocol. Primers were designed using the Universal Probe Library for Human (Roche Applied Science, Roche, Penzberg, Bavaria, Germany) and used at a final concentration of 20 μM. The sequences of the forward and reverse primers used are as follows: for p21, 5′-cgaagtcagttccttgtggag-3′ and 5′-catgggttctgacggacat-3′; and for β-actin, 5′-ccaaccgcgagaagatga-3′ and 5′-ccagaggcgtacagggatag-3. Real-time quantitative PCR was performed in the Bio-Rad iQ5 system using a thermal profile of an initial 5-min melting step at 95 °C, followed by 40 cycles at 95 °C for 10 s and 60 °C for 60 s. Relative mRNA levels of the genes of interest were normalized to β-actin expression using the simplified comparative threshold cycle delta CT method [2−(ΔCT gene of interest −ΔCT β-actin].

### Trypan Blue Staining

Cell suspension was mixed with 0.4 % (*w*/*v*) Trypan Blue solution (Sigma-Aldrich), and the number of live cells was determined using a hemocytometer. Cells that failed to exclude the dye were considered nonviable.

### MTT Assay

This assay is based on the ability of viable cells to reduce yellow MTT to blue formazan. Cells were seeded at an initial concentration of 1 × 10^6^ cells × mL^−1^ on 96-well plates. After 24 h of treatment with 30 mM 2-deoxy-D-ribose and/or α-pifithrin cells were incubated with 20 μl of MTT (thiazolyl blue tetrazolium bromide (1 mg/mL, 3 h) and subsequently solubilized in dimethyl sulfoxide (DMSO). The extent of reduction of MTT was quantified by absorbance measurement at 595 nm with 630 nm as a reference wavelength, according to the manufacturer’s protocol. Cell survival was estimated as the percentage of the value of untreated controls. All reagents were purchased from Sigma-Aldrich.

### Confocal Laser Scanning Microscopy

Cells were fixed for 15 min in 4 % paraformaldehyde, washed in PBS/BSA, and cytospun at 700 rpm for 7 min onto poly-L-lysine-coated slides. Then they were blocked and permeabilized with 0.5 % Triton X-100 in PBS-0.5 % BSA for 20 min at room temperature and incubated with rabbit anti-p21 antibody (1:500). After washing with PBS, cells were incubated with Alexa Fluor 488-conjugated anti-rabbit antibody. The preparations were mounted on ProLong® Gold Antifade Reagent with DAPI (Thermo Fisher) and visualized with LEICA TCS-SP5-AOBS confocal microscope system (Heidelberg, Germany).

### Flow Cytometry Cell Cycle Analysis

Distribution of cells in SubG1, S, and G2/M cell cycle phases was measured based on propidium iodide (PI) staining using standard flow cytometry method. Cell lines were seeded at an initial concentration of 1 × 10^6^ cells mL^−1^ and incubated for 24 h with or without 30 mM 2-deoxy-D-ribose. Exponentially growing cells were used in a single cell cycle measurement performed in triplicate with fluorescence-activated cell sorting (FACS). After centrifugation (400×*g*, 4 °C, 5 min), cells were washed with PBS, suspended in 70 % cold ethanol for 1 h, washed again and incubated for 1 h in 0.1 % sodium citrate in PBS containing RNase (10 μg/mL) and 50 μg/mL PI (Sigma-Aldrich). Measurements were performed using FACSCalibur instrument (Becton Dickinson, Franklin Lakes, NJ, USA). Prior to analysis, cells were equilibrated to room temperature. Exactly 10,000 events from each sample were collected in a single cell gate. Aggregates and debris were excluded from analysis by creating the gate on the FL2-W (transit time) versus FL2-A (total cell fluorescence) cytogram according to the standard procedure [77]. The percentages of cells in SubG1 phase were determined based on the frequency distribution of DNA content with ModFit LT software (version 3.1, Verity Software House, Topsham, Maine).

### Annexin V Assay

Apoptosis was measured by flow cytometry using Annexin V-FITC (Sigma-Aldrich) and propidium iodide (Sigma-Aldrich) staining. Cell lines were seeded at an initial concentration of 1 × 10^6^ cells mL^−1^. After 24 h cells were collected, centrifuged (400×*g*, 4 °C, 5 min) and washed with 1 ml PBS. Afterward cells were incubated 15 min in the dark and on ice in 100 μl Annexin V incubation buffer (0.1 M Hepes, 25 mM CaCl2, 1.4 M NaCl) with 1 μl Annexin V-FITC. After incubation 400 μl Annexin binding buffer with 2 μl PI (50 μg/ml) were added. By staining cells with a combination of Annexin V-FITC and PI, it is possible to distinguish and quantitatively analyze non-apoptotic cells (Annexin V-FITC negative/PI negative), early apoptotic cells (Annexin V-FITC positive/PI negative), late apoptotic/necrotic cells (Annexin V-FITC positive/PI positive), and dead cells (Annexin V-FITC negative/PI positive). Measurements were performed using FACSCalibur instrument (Becton Dickinson) and data were analyzed with BD Cell Quest Pro Software.

### Mitochondrial Membrane Potential (MMP) Assessment

To assess MMP, staining with cationic dye JC-1 was performed. Cells were seeded at a density of 1 × 10^6^ cells mL^−1^ and treated with or without 30 mM 2-deoxy-D-ribose. After 24 h cells were collected, centrifuged (400×*g*, 4 °C, 5 min) and washed twice with PBS. After washing, cells were incubated for 20 min in the incubator (37 °C, 5%CO_2_) with 10 μl JC-1 staining solution (MitoProbe Jc-1 Assay Kit, Invitrogen) diluted in 1 ml PBS. Healthy cells with functional mitochondria containing red JC-1 aggregates are detectable in FL-2 channel. Apoptotic or unhealthy cells with collapsed mitochondria contain mainly green JC-1 monomers and are detectable in FL-1 channel. As a positive control we used carbonyl cyanide m-chlorophenyl hydrazone (*CCCP)*, which is a chemical inhibitor of oxidative phosphorylation and causes an uncoupling of the proton gradient. Measurements were performed using FACSCalibur instrument (Becton Dickinson). Analysis was made with BD Cell Quest Pro Software.

### Statistics

Statistical analyses were performed with Graph Pad Prism 6 (La Jolla, CA, USA). All the statistical data are presented as mean ± standard error of the mean (SEM). Normality was checked with the Shapiro–Wilk test. Parametric tests were therefore used in the statistical analysis. Based on the expertise achieved on previous works we can expect that, with the sample size we used and the significance level we fixed, the variability within groups will be low enough and the differences between groups to detect will be high enough to ensure a statistical power above 0.9. Statistical significance was estimated by the Student’s *t* test or, when appropriated, by one-way and two-way analysis of variance (ANOVA) followed by the Tukey’s test for multiple comparisons. A value of *p* < 0.05 was considered significant.
